# De Novo Transcriptome Assembly and Comparative Analysis of Differentially Expressed Genes Involved in Cold Acclimation and Freezing Tolerance of the Arctic Moss *Aulacomnium turgidum* (Wahlenb.) Schwaegr

**DOI:** 10.3390/plants12061250

**Published:** 2023-03-09

**Authors:** Pilsung Kang, Yo-Han Yoo, Dong-Il Kim, Joung Han Yim, Hyoungseok Lee

**Affiliations:** 1 Division of Life Sciences, Korea Polar Research Institute, Incheon 21990, Republic of Korea; lovesong3233@kopri.re.kr (P.K.); yohan04@korea.kr (Y.-H.Y.);; 2 Department of Biological Engineering, Inha University, Incheon 22212, Republic of Korea; 3 Polar Science, University of Science and Technology, Incheon 21990, Republic of Korea

**Keywords:** *Aulacomnium turgidum*, cold acclimation, de novo assembly, freezing stress, RNA-seq

## Abstract

Cold acclimation refers to a phenomenon in which plants become more tolerant to freezing after exposure to non-lethal low temperatures. *Aulacomnium turgidum* (Wahlenb.) Schwaegr is a moss found in the Arctic that can be used to study the freezing tolerance of bryophytes. To improve our understanding of the cold acclimation effect on the freezing tolerance of *A. turgidum*, we compared the electrolyte leakage of protonema grown at 25 °C (non-acclimation; NA) and at 4 °C (cold acclimation; CA). Freezing damage was significantly lower in CA plants frozen at −12 °C (CA-12) than in NA plants frozen at −12 °C (NA-12). During recovery at 25 °C, CA-12 demonstrated a more rapid and greater level of the maximum photochemical efficiency of photosystem II than NA-12, indicating a greater recovery capacity for CA-12 compared to NA-12. For the comparative analysis of the transcriptome between NA-12 and CA-12, six cDNA libraries were constructed in triplicate, and RNA-seq reads were assembled into 45,796 unigenes. The differential gene expression analysis showed that a significant number of AP2 transcription factor genes and pentatricopeptide repeat protein-coding genes related to abiotic stress and the sugar metabolism pathway were upregulated in CA-12. Furthermore, starch and maltose concentrations increased in CA-12, suggesting that cold acclimation increases freezing tolerance and protects photosynthetic efficiency through the accumulation of starch and maltose in *A. turgidum*. A de novo assembled transcriptome can be used to explore genetic sources in non-model organisms.

## 1. Introduction

Mosses are one of the oldest land plants and are spread all around the world [1]. They typically inhabit humid environments like rainforests, wetlands, and alpine ecosystems. However, they are also distributed in dry and cold polar regions [2]. *Aulacomnium turgidum* (Wahlenb.) Schwaegr is a moss widely found above the Arctic Circle, including in Svalbard and Alaska, with extreme climates [3]. It has been reported to be capable of tissue regeneration after 400 years of ice entombment [4], suggesting that it has a unique mechanism for freezing tolerance. In previous studies, its transcriptome information was revealed through the construction and analysis of a small-scale complementary DNA (cDNA) library [5], and a complete mitogenome sequence of *A. turgidum* has been reported [6]. However, even though *A. turgidum* is suitable for studying freezing tolerance, our understanding of *A. turgidum* remains insufficient.

Due to their sessile lifestyle, plants must cope with abiotic stresses such as cold, heat, salinity, and drought. Freezing stress is a significant challenge for plants to survive [7]. When exposed to freezing environments, organelles and cell membranes are physically damaged by ice crystals and osmotic stress occurring during the freezing process [8]. In addition, reactive oxygen species (ROS) such as singlet oxygen (^1^O_2_), superoxide (O_2_∙), hydroxyl radical (OH∙) and H_2_O_2_ are excessively generated and accumulated by electrons leaked from physically damaged mitochondria [9]. Enzymes, DNA, and unsaturated fatty acids are then denatured by accumulated ROS [10], which leads to increased cell damage and, ultimately, cell death [11].

Plant cells exposed to freezing stress can increase their tolerance using various cellular compounds and molecular events. These include anti-freeze compounds, osmoprotectants (proline, sucrose, fructan, mannitol, and glycine betaine), secondary messenger (calcium ion flux), and cold-regulated (COR) genes, including the LEA family [10,12]. For example, when plant cells are exposed to freezing-induced dehydration, COR15a protein increases the cryostability of the plasma membrane, which helps increase cell survival even at low freezing temperatures [13,14]. Fructan acts as an anti-freezing agent protecting cells by lowering the freezing point of water in the cells [15]. Proline, fructan, and glycine betaine play a role in regulating osmosis under osmotic stress due to dehydration, and they also have enzyme degradation inhibition and antioxidant effects [10,16]. The cytosolic calcium concentration of plant cells increases as plants are exposed to cold stress. This triggers phosphorylation cascades through calcium-binding proteins (CBPs) interacting with other proteins, ultimately leading to cold stress resistance. Interestingly, some species are able to increase freezing tolerance after exposure to low non-freezing temperatures, termed cold acclimation [17,18].

Genome-wide transcriptome analysis aids in understanding protective cellular responses. Quantification and comparison of transcript expression are widely used in molecular biology research. In particular, RNA-Seq technology and de novo transcriptome assembly are utilized to reconstruct and quantify whole transcriptomes in non-model organisms without a reference genome [19]. These technological advances can be employed to assess differential gene expression for non-model organisms and explore genetic diversity. Although mosses comprise a large phylum containing 13,000 species [20], only 21 genomes from eight genera have been reported, including model species, Antarctic moss, and peat mosses [21,22,23,24]. Additionally, RNA-seq-based de novo transcriptome studies have been reported in several non-seed plants, including *Funaria hygrometrica* (habitat: nutrient-rich soils including old bonfire sites) [25], *Bryum argenteum* (habitat: very dry and usually rich in nutrients such as nitrates) [26], *Ceratodon purpureus* (habitat: common and cosmopolitan in healthland and grassland) [27], *Leptobryum pyriforme* (habitat: easily found as a weed in glasshouses) [28], and *Dicranum scoparium* (habitat: frequent on the ground in woodland, and trees and logs) [29]. In 2019, the 1KP Plants project released transcriptomes of 1173 plant species, including 41 mosses [30]. However, there is still a need to accumulate more genomic resources through genome-wide transcriptome analysis of various moss species.

**Figure 1:**
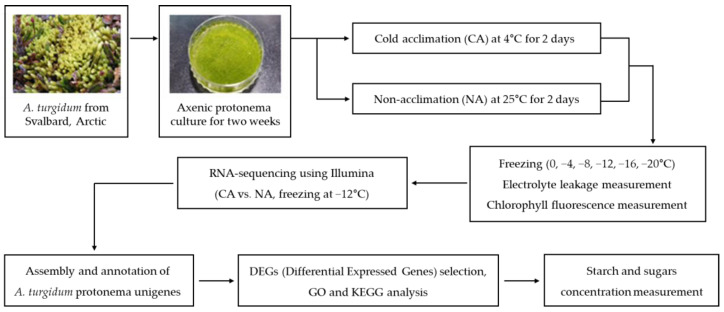
Workflow diagram summarizing the analysis process used in this study.

In the present study, we prepared two groups of axenic protonema of *A. turgidum,* with and without cold acclimation. After freezing stress treatment, we aimed to investigate transcriptome changes that mediate the cold acclimation process. To this end, we obtained Illumina technology-based RNA sequence data and created a transcriptome reference for *A. turgidum* through de novo assembly. Differentially expressed genes were selected from the cold acclimation treatment sample, and information about their biological functions was provided. Finally, we measured the concentration of starch and sugars to determine whether cold acclimation affects carbohydrate metabolism. The overall process for this study is summarized in Figure 1.

## 2. Results

### 2.1. Physiological Changes Due to Cold Acclimation Treatment under Freezing Stress

**Figure 2:**
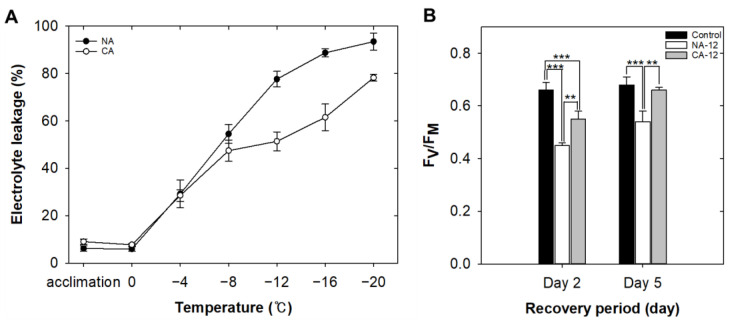
Physiological changes of *A. turgidum* protonema induced by cold acclimation and freezing stress. Freezing stress was applied to *A. turgidum* protonema at a rate of decreasing the temperature of 2 °C per hour. Electrolyte leakage change under freezing stress for CA and NA (**A**). The maximum photochemical efficiency of photosystem II (F_V_/F_M_) values of CA-12 and NA-12 during recovery from freezing (**B**). Data are means ± SD of three repeats (***, *p* < 0.001; **, *p* < 0.01, Student *t*-test). CA, cold-acclimated *A. turgidum* at 4 °C for 2 days; NA, non-acclimated *A. turgidum* at 25 °C for 2 days; CA-12, CA exposed to freezing until −12 °C; NA-12, NA exposed to freezing until −12 °C.

As electrolyte leakage (EL) is considered a good indicator of cell membrane integrity [31], we performed an electrolyte leakage assay to compare membrane integrity and freezing sensitivity of *A. turgidum* protonema with cold acclimation (CA) and without (non-acclimation; NA). The protonema of *A. turgidum* cultured at 25 °C for two weeks was transferred to a fresh BCDAT agar plate and acclimated at 4 °C (CA) and 25 °C (NA) under continuous light for 48 h. Then, changes in EL of protonema of *A. turgidum* at different sub-zero temperatures (0 to −20 °C) were examined. When the 50% electrolyte leakage (EL_50_) of the NA and CA were compared, the temperature of CA EL_50_ (−10.6 °C) was 3.3 °C lower than the temperature of NA EL_50_ (−7.3 °C). Furthermore, the greatest difference in EL of CA and NA was observed at −12 °C (Figure 2A).

Protonema of both CA and NA exposed to freezing at −12 °C were designated as CA-12 and NA-12, respectively. To assess the effect of freezing on the photosynthetic capacity of *A. turgidum*, they were transferred to the 25 °C culture room for recovery. Immediately after freezing exposure, F_V_/F_M_ values of CA-12 and NA-12 were 0.28 ± 0.07 and 0.19 ± 0.02, respectively. On the second day of recovery, the value of CA-12 was higher than that of NA-12 and lower than that of the unfrozen control. By the fifth day of recovery, the value of CA-12 (0.66 ± 0.01) had recovered to the same level as the control (0.69 ± 0.01), whereas NA-12 (0.54 ± 0.04) had only recovered to 79.5% of the control (Figure 2B). This result suggested that cold acclimation resulted in physiological changes in the protonema of *A. turgidum* under freezing stress. Therefore, RNA from CA-12 and NA-12 samples was isolated for RNA sequencing.

### 2.2. De Novo Assembly and Annotation of Assembled A. turgidum Protonema Transcripts

To comprehensively understand the transcriptome profile related to enhanced freezing tolerance due to cold acclimation, NA and CA plants were sampled in triplicate after treatment at −12 °C and then sequenced. The total number of raw reads from six libraries was 21,339,944, and 19,990,976 trimmed reads were generated using Trimmomatic (i.e., 93.65% of trimmed rate; Table 1). De novo assembly was carried out using Trinity, and 116,979 contigs and 45,796 unigenes with N50 (bp) values of 2481 and 1017 were obtained, respectively (Table 2).

**Figure 3:**
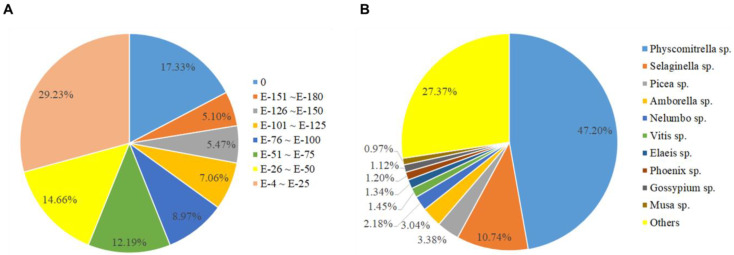
E-value distribution of the top five BLASTx hits for each unigene (less than 1.0 × 10^−4^) (**A**) and Nr annotation species distribution analysis (**B**).

Functional annotation of each unigene was obtained by BLASTx against the non-redundant protein database (Nr). The similarity analysis of the Nr database revealed that 56.12% of unigenes had a significant homology (E-value < 1 × 10^−50^) (Figure 3). The Nr annotation species distribution analysis revealed that a majority of *A. turgidum* unigenes had a significant match to *Physcomitrella* sp. (47.20%), followed by *Selaginella* sp. (10.74%), *Picea* sp. (3.38%), *Amborella* sp. (3.04%), *Nelumbo* sp. (2.18%), *Vitis* sp. (1.45%), *Elaeis* sp. (1.34%), *Phoenix* sp. (1.20%), *Gossypium* sp. (1.12%), *Musa* sp. (0.97%), and others (27.37%). Because of research into *A. turgidum* has been rarely carried out. Only three (0.0040%) unigenes were annotated in the Nr database against *A. turgidum* (Figure 3, Table S1).

### 2.3. Gene Ontology (GO) Distribution and KEGG Pathway Analysis Using Whole Unigenes

The GO distribution of the *A. turgidum* unigenes was analyzed. A total of 10,228 (22.33%) unigenes were assigned to at least one GO term, and 6992, 4965, and 6375 unigenes were classified into the groups of “biological processes”, “molecular functions”, and “cellular components”, respectively (Table S2). Within the “biological processes” category, “cellular processes”, “metabolic processes”, and “single-organism processes” were the most abundant sub-categories. In the “molecular functions” category, “binding” and “catalytic activity” sub-categories made up the main proportion of the unigenes. In the sub-category of “cellular components”, “cell parts”, “cells”, “membranes”, and “organelles” were abundant (Table S2). In addition, we performed KEGG pathway annotations with the acquired unigenes to gain insight into the molecular interactions, reactions, and relationship networks of *A. turgidum.* 2171 (4.74%) unigenes were assigned to at least one KEGG pathway annotation into 42 KEGG pathways. They were identified in five categories: “metabolism”, “genetic information processing”, “environmental information processing”, “cellular processes”, and “organismal systems”. The most abundant categories in the KEGG Orthology hierarchies were “ribosomes”, “protein processing in the endoplasmic reticulum”, and “spliceosome” (Table S3).

### 2.4. RNA-Seq Analysis Identified Candidate Unigenes Associated with Cold Acclimation and Freezing Stress

**Figure 4:**
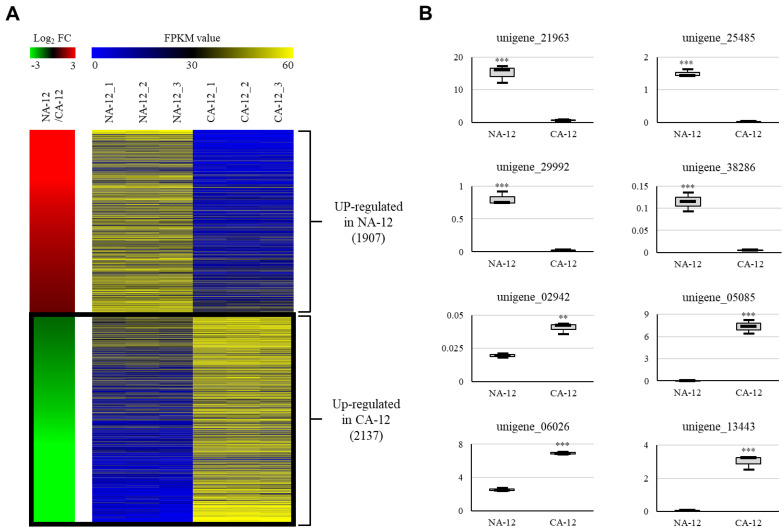
Heatmap of differentially expressed unigenes of CA-12 compared to NA-12. Using RNA-seq data analysis under the criteria of *p*-value < 0.05 and a log_2_ ratio of <−1 or >1 of NA-12 versus CA-12, we identified 4044 differentially expressed unigenes (**A**). In the left panel, red indicates upregulation in NA-12; green indicates upregulation in CA-12. The right panel shows average normalized FPKM values from RNA-seq experiments; blue indicates the lowest expression level, and yellow is the highest. Detailed data from the RNA-seq analysis are presented in Table S4. The association with the heatmap was further confirmed by monitoring eight genes using qPCR (**B**). The *y*-axis indicates the expression level relative to EF1/unigene_08701 (internal control), and the *x* axis indicates samples used for qPCR. Data are means ± SD of three repeats (***, *p* < 0.001; **, *p* < 0.01, Student *t*-test). The primer sequences used for qPCR analysis are shown in Table S5. CA-12, *A. turgidum* protonema were cold-acclimated at 4 °C for 2 days and exposed to freezing until −12 °C; NA-12, *A. turgidum* protonema were non-acclimated at 25 °C for 2 days and exposed to freezing until −12 °C.

To identify the genes showing differential expression patterns by cold acclimation in *A. turgidum*, we compared CA-12 and NA-12 with three biological replicates. In CA-12, there were 2137 unigenes upregulated and 1907 unigenes downregulated when compared with NA-12 (*p*-values < 0.05 and |log_2_ fold-change| > 1) (Figure 4A, Table S4). To verify the DEGs, we selected four unigenes (unigene_02942, unigene_05085, unigene_06026, and unigene_13443) that were upregulated and four (unigene_21963, unigene_25485, unigene_29992, and unigene_38286) that were downregulated in CA-12. Then, we confirmed the expression patterns of the eight unigenes by real-time quantitative PCR (qPCR). The RNA-Seq data showed a significant positive correlation with the qPCR data (Figure 4B), verifying that DEGs selected through RNA-Seq analysis had high accuracy.

### 2.5. Transcription Factors Analysis of the Upregulated Group in the CA-12 Treatment

**Figure 5:**
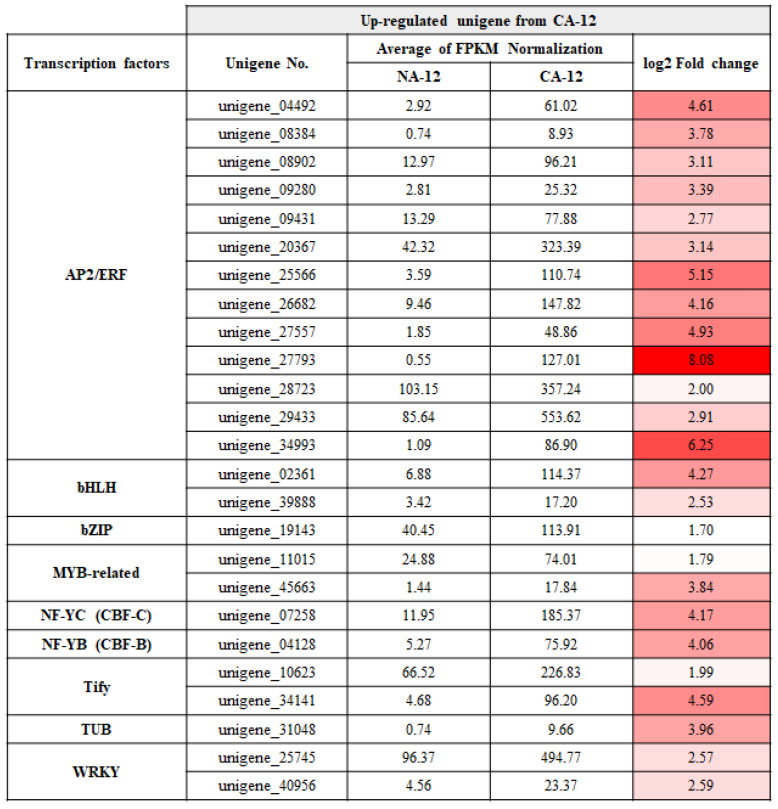
List of up-regulated unigenes encoding transcription factors in the CA-12 treatment. The average FPKM normalization and log_2_ fold change values for the identified 25 unigenes are described. In the log_2_ fold change column, red indicates the highest value, and white indicates the lowest value. CA-12, *A. turgidum* protonema were cold-acclimated at 4 °C for 2 days and exposed to freezing until −12 °C; NA-12, *A. turgidum* protonema were non-acclimated at 25 °C for 2 days and exposed to freezing until −12 °C.

Transcription factors (TFs) are major regulators that control gene expression by binding to specific DNA sequences. We identified 25 upregulated unigenes encoding TFs out of 2137 unigenes upregulated by cold acclimation followed by freezing. These included 13 APETALA2/ethylene responsive factor (AP2/ERF) TFs (unigene_04492, unigene_08384, unigene_08902, unigene_09280, unigene_09431, unigene_20367, unigene_25566, unigene_26682, unigene_27557, unigene_27793, unigene_28723, unigene_29433, and unigene_34993), two basic helix-loop-helix (bHLH) TFs (unigene_02361 and unigene_39888), two MYB TFs (unigene_11015 and unigene_45663), two WRKY TFs (unigene_25745 and unigene_40956), and 6 other upregulated TF encoding unigenes under CA-12 conditions (Figure 5). These results indicate that AP2/ERF are likely pivotal regulators for the enhanced freezing tolerance due to cold acclimation in *A. turgidum*.

### 2.6. GO Distribution and KEGG Pathway Analysis of the Upregulated Group in CA-12

**Figure 6:**
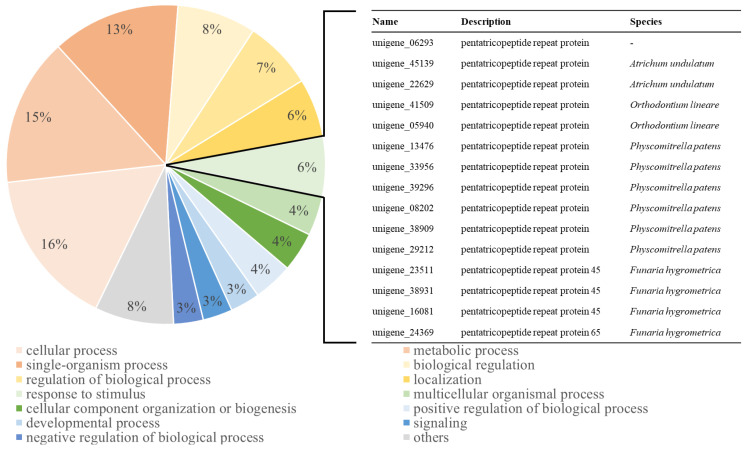
Gene ontology (GO) analysis in the “biological processes” category for unigenes upregulated in response to CA-12 treatment. Thirteen GO terms were over-represented, with the rest collectively indicated as “others”. In the circle graph, the percentage occupied by each GO term is expressed as “%”. Fifteen unigenes encoding pentatricopeptide repeat (PPR) protein were identified in “response to stimulus” (6%), which is a distinguished feature for *A. turgidum*. Details of GO information for groups upregulated in CA-12 treatment are presented in Table S6.

To identify the biological functions of the 2137 unigenes upregulated by CA-12 treatment, we performed a GO term analysis of these genes in the “biological processes” category. This revealed that 16% related to “cellular process”, 15% to “metabolic process”, 13% to “single-organism process”, 8% to “biological regulation”, and 7% to “regulation of biological processes”. In addition, the sub-category “response to stimulus” (6%) was significantly associated with cold acclimation (Figure 6, Table S6). Interestingly, the pentatricopeptide repeat protein was most commonly found in this category along with “ATP-binding cassette transporter”, “lipoxygenase”, and “cellulose synthase” (Table S6).

Subsequent KEGG pathway analysis (corrected *p*-Value < 0.01; Table 3) of these 2137 upregulated unigenes identified 22 pathways, 21 of which were included in the “metabolism” category. Interestingly, 10 of the 21 subcategories were associated with carbohydrate metabolisms, such as starch and sucrose metabolism (ppp00500; 32 unigenes), glycolysis/gluconeogenesis (ppp00010; 26 unigenes), amino sugar and nucleotide sugar metabolism (ppp00520; 23 unigenes), the pentose phosphate pathway (ppp00030; 16 unigenes), galactose metabolism (ppp00052; 13 unigenes), fructose and mannose metabolism (ppp00051; 12 unigenes), and others (Table 3 and Table S7). These results suggest a close relationship between cold acclimation and carbohydrate metabolism, which could potentially affect the freezing tolerance of *A. turgidum*.

### 2.7. Differences in Starch and Sugar Concentrations According to Cold Acclimation and Freezing Stress

**Figure 7:**
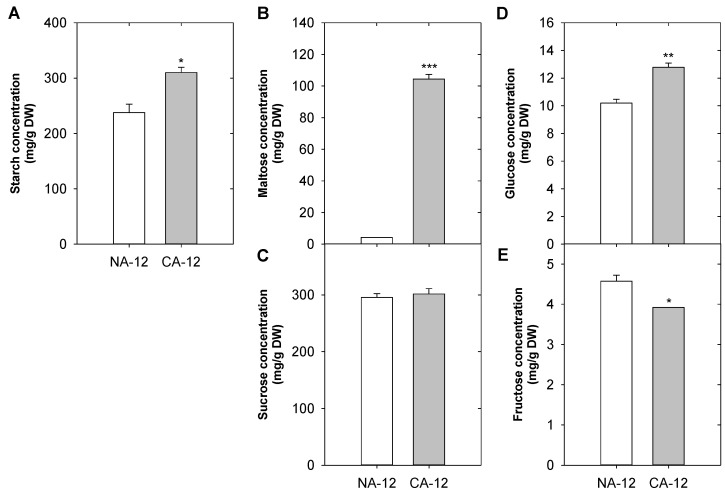
Concentration changes of starch and sugars in *A. turgidum* protonema of NA-12 and CA-12. Starch (**A**), Maltose (**B**), Sucrose (**C**), Glucose (**D**) and Fructose (**E**). The *y*-axis scales of each graph are different. CA-12, *A. turgidum* protonema were cold-acclimated at 4 °C for 2 days and exposed to freezing until −12 °C; NA-12, *A. turgidum* protonema were non-acclimated at 25 °C for 2 days and exposed to freezing until −12 °C. Data are means ± SD of three repeats (***, *p* < 0.001; **, *p* < 0.01; *, *p* < 0.05, Student *t*-test).

To determine if cold acclimation affects carbohydrate metabolism, we measured the starch concentrations and detected a higher starch content in CA-12 than in NA-12 (Figure 7A). In addition, concentrations of maltose, sucrose, glucose, and fructose were measured using HPLC. The content of maltose and glucose in CA-12 was significantly higher than that of NA-12. However, the fructose content was lower in CA-12, and there was no significant difference in sucrose between NA-12 and CA-12 (Figure 7B–E). These results suggest that cold acclimation in *A. turgidum* increases tolerance to freezing stress through the accumulation of starch, maltose, and glucose.

## 3. Discussion

Although it is an extreme environment, many plant species live in the Arctic. Several ecological studies have reported that these plants have evolved in the direction of acquiring a freezing tolerance mechanism to adapt to extreme environments [2,32,33,34]. Still, explanations using physiological and molecular biological analysis analyses are limited.

### 3.1. Enhanced Freezing Tolerance by Cold Acclimation

Freezing can decrease photosynthetic efficiency by damaging chloroplasts, and the F_V_/F_M_ ratio serves as an indicator to easily measure the degree of thylakoid membrane damage [35]. Researchers have found significant differences among winter wheat cultivars in terms of F_V_/F_M_ recovery from freezing [36]. They observed that the degree of recovery of F_V_/F_M_ in sweet cherry leaves depends on the number of freezing events experienced [37]. We assessed the level of freezing tolerance in *A. turgidum* protonema both before and after cold acclimation using a combination of electrolyte leakage and chlorophyll fluorescence measurement. The results indicate that CA-12 has an enhanced tolerance to freezing and a greater capacity for photosynthetic recovery than NA-12, showing more rapid and higher F_V_/F_M_ recovering rates (Figure 2). Furthermore, we performed RNA-sequencing to generate transcriptome reference of *A. turgidum* and selected unigenes whose expression increased following cold acclimation under freezing stress. The transcriptome acquired in this study provides insight into the gene regulatory network of Arctic mosses and other non-seed plants for freezing stress tolerance mechanisms.

### 3.2. AP2/ERF Family Transcription Factors Are Predicted to Contribute to Freezing Stress Tolerance of A. turgidum

Transcription factors (TFs) are important regulators that activate or suppress gene expression to modulate signal transduction and play a pivotal role in plant development, cell signaling, and stress response [38]. TFs regulate downstream target gene expression by binding to cis-acting elements in the promoter region [39]. In this research, we identified 2137 unigenes that responded to cold acclimation under freezing stress, of which 25 were classified as TFs (Figure 5). Various major TF families, such as AP2/ERF, MYB, NAC, and WRKY, contribute to improved plant resistance in response to various stimuli [40]. Interestingly, 13 ou 25 TFs belonged to the AP2/ERF family (Figure 5). The AP2/ERF family proteins have an AP2/ERF DNA-binding domain that interacts directly with GCC box and/or dehydration-responsive element (DRE)/C-repeat element (CRT) cis-acting elements of the promoter [41]. This family can be further divided into five subfamilies, depending on the number and similarity of DNA-binding domains: AP2 (APETALA2), RAV (related to ABI3/VP1), DREB (dehydration-responsive element binding protein), ERF (ethylene-responsive factor), and others [42]. Among these subfamilies, the DREB-A1 subgroup, including several C-Repeat-Binding Factors (CBFs), is known to increase freezing stress tolerance. CBF activates DRE containing Cold Responsive Genes (CORs), along with the Inducer of CBF Expression (ICE) [43]. CORs encode the Late Embryogenesis Abundant (LEA) protein which enables resistance to cold stress in plants by modifying sugar metabolism and fatty acid desaturation [44]. Studies on the improvement of cold stress tolerance by DREBs have been conducted in various plants: *TaDREB1* in wheat [45], *BpERF13* in birch [46], and *OsDREB1G* in rice [47]. For example, expression of the *DREB1A* gene in *Arabidopsis thaliana* was induced by low-temperature stress, and transgenic plants overexpressing *DREB1A* showed enhanced tolerance to freezing and dehydration [48]. In rice, the *OsDREB1A* and *OsDREB1B* expression was induced by cold stress, and overexpression of *OsDREB1A* in transgenic *Arabidopsis* led to higher tolerance to drought, high salt, and freezing stresses [49]. Furthermore, AP2/ERF TFs have been known to protect plants from multiple stresses by the action of various plant hormones, protein interaction partners, and plant epigenetics, such as DNA methylation and histone modification [50,51]. Thus, the role of the AP2/ERF family TFs may be one of the reasons why *A. turgidum* can survive in the Arctic region. The 13 AP2/ERF unigenes presented in Figure 5 can be used to understand the biological processes that *A. turgidum* undergoes to acquire tolerance to freezing stress.

### 3.3. AP2/ERF Family TFs Are Associated with Pentatricopeptide Repeat (PPR) Proteins and Carbohydrate Metabolism

Based on GO and KEGG analysis of upregulated unigenes in CA-12, we found that pentatricopeptide repeat (PPR) proteins and sugar metabolism were enriched by cold acclimation and freezing (Figure 6; Table 3). PPR proteins are RNA-binding proteins characterized by tandem arrays of a degenerate 35-amino-acid (PPR motifs) [52]. The PPR protein not only participates in post-transcriptional processes, including RNA editing, splicing, stability, cleavage, degradation, and translation but also appears to play an important role in response to abiotic stresses [53,54]. PPR proteins constitute a large family of land plants—with 450 members in *Arabidopsis* and 477 in rice, while the moss *Physcomitrella patens* have only 105 PPR genes [55,56]. Interestingly, PPR proteins are involved in the expression of AP2/ERF TFs. For example, GUN1, a DNA-binding chloroplast PPR protein, regulates the expression of *ABI4* (*ABSCISIC ACID INSENSITIVE-4*), one of the AP2/ERF TFs [57]. In addition, the cytosol-nucleus dual-localized PPR protein SOAR1 is known to regulate the expression of cold-responsive genes in the C-repeat binding factor/DRE-binding factor (CBF/DREB) transcriptional regulatory cascade. *SOAR1*-overexpression lines were reported to increase the expression of *CBF1/DREB1B*, *CBF2/DREB1C*, and *CBF3/DREB1A,* as well as CBF downstream regulon genes *COR15A*, *COR15B*, *COR414,* and *KIN1,* during cold stress [58]. This implies that PPR might influence the acquisition of tolerance to freezing stress by regulating the expression of AP2/ERF TFs.

The AP2/ERF family also participates in carbohydrate metabolism. For instance, the galactinol synthase-encoding gene has CRT/DRE regulatory elements in its promoter region and is highly expressed in *CBF*-overexpressing Arabidopsis and rice [59,60]. In contrast, Arabidopsis *GOLS3* in *cbf123-1* triple mutants was reduced by about 55-fold, and the expression of 28 other genes involved in carbohydrate metabolisms, such as sucrose synthase and β-glucosidases, was also significantly decreased [44]. In addition, *SUN6* (*sucrose uncoupled-6*)/*ABI4* mutation has been reported to be insensitive to sugars, a substrate of hexokinase. These findings suggest that *SUN6/ABI4* may play a role in the hexokinase-dependent sugar responses [61]. There have been multiple reports that freezing tolerance is improved by AP2/ERF [43,62] and soluble sugars [63,64]. We further verified that the concentrations of starch and maltose increased, and various unigenes related to sugar metabolism were upregulated by cold acclimation (Figure 7 and Figure S1). Taken together, the role of AP2/ERF family TFs is presumed to be one of the reasons for this result.

### 3.4. Starch and Soluble Sugars Play an Important Role in Freezing Stress Tolerance

Starch is the major storage unit of carbohydrates and the primary products of photosynthesis in plants. Recently, its importance in energy metabolism, developmental processes, and temperature acclimation has been recognized [65]. Interestingly, the role of starch in adaptation to low temperatures varies between different plant species [66]. For instance, Arabidopsis increases the content of glucose, fructose, sucrose, maltose, and starch under cold stress (4 °C) [67]. In this study, *A. turgidum* CA plants whose freezing tolerance was increased due to cold acclimation also had higher starch content than control NA plants. On the other hand, *Physcomitrella patens* was reported to have reduced starch content at low temperatures (−16 °C to 4 °C) [68]. These results confirm that the dynamics of synthesis and breakdown pathways play a more pivotal role than the absolute amount of starch under abiotic stresses [69].

Accumulation of soluble sugars correlates with the stabilization of biological components under cold stress [70]. Maltose, a disaccharide produced through the decomposition of starch by beta-amylase (βAM) [64], serves as an intermediate component when starch provides hexose to synthesize sucrose or when it is broken down into glucose [71,72]. Under cold stress, photosynthesis is maintained by maltose as it helps to protect chloroplasts from the osmotic stress caused by dehydration [69]. Freezing tolerance was enhanced by increasing maltose metabolism in *MAL62* (a maltase encoding gene) overexpressing yeast [73]. Glucose is a well-known substance to assist in the regulation of intracellular osmotic pressure [74]. The higher glucose content of cold-tolerant *Pinus halepensis* is associated with increased cold tolerance compared to cold-sensitive *P. halepensis* [75]. This is in accordance with changes in the sugar contents of *A. turgidum* CA-12 plants observed in the current study. Starches, maltose and glucose concentrations may have increased in cold-acclimated *A. turgidum* plants due to the enhanced transcription of unigenes associated with the synthesis, decomposition, conversion, and transport of starch, maltose, sucrose, glucose, and fructose among the 2137 unigenes which were upregulated when the plants were exposed to freezing stress (Figure S1).

Plants are exposed to both biotic (insect herbivores and microbial pathogens) and abiotic (extreme temperature and inappropriate water supply) stresses throughout their lifetimes. Acclimation is a key strategy employed to survive these environmental stresses. Priming is, like acclimation, the strategy of remembering the first biotic attack to respond effectively to the second attack [76]. Research has shown that a “primed” state can increase defence responses and enhance resistance to stress [77]. An analysis of expression changes of unigenes linked to cold acclimation in *A. turgidum* is expected to provide biological information on molecular strategies of polar mosses to defend against various external stimuli.

## 4. Materials and Methods

### 4.1. Sample Preparation

*A. turgidum* specimens were collected in the vicinity of the Korean Dasan Arctic Station (78°54′ N/11°57′ W) near Ny-Ålesund, Svalbard, in August 2006. A small patch of *A. turgidum* was washed using 30 mL 1.2% NaOCl containing 1 drop Tween-80 at 180 rpm for 15 min, then washed twice with sterilized water. Axenic *A. turgidum* was cultured in BCDAT medium containing 250 mg/L KH_2_PO_4_, 250 mg/L MgSO_4_, 12.5 mg/L FeSO_4_·7H_2_O, 920.5 mg/L ammonium tartrate, 1010 mg/L KNO_3_, 147 mg/L CaCl_2_·2H_2_O, 0.614 mg/L H_3_BO_3_, 0.389 mg/L MnCl_2_·4H_2_O, 0.055 mg/L CuSO_4_·5H_2_O, 0.055 mg/L CoCl·6H_2_O, 0.055 mg/L ZnSO_4_·7H_2_O, 0.028 mg/L KI, 0.025 mg/L Na_2_MoO_4_·2H_2_O, and 0.8% phytoagar at pH 5.8.

### 4.2. Cold Acclimation and Freezing Stress Treatment

The two-week-old *A. turgidum* protonema grown on fresh BCDAT agar plates were transferred to a 4 °C cold room (cold acclimation; CA), and the others were left in the 25 °C culture room (non-acclimation; NA) under continuous light for 2 days. The refrigerated circulating water bath program and freezing stress treatment were carried out as described [78,79]. Briefly, 100 mg (dry weight) protonema from each of the CA and NA groups were transferred to tubes and rinsed carefully three times using deionized water (DW). Then, 100 μL DW was added to each tube. The tubes were placed in the refrigerated circulating bath (Gaon Science Instrument, South Korea) with the temperature preset at 0 °C for 30 min. Tiny pieces of ice were added to each tube for ice nucleation, and the circulating bath was programmed to cool at a rate of −2 °C per hour. The tubes were removed from the bath when the designated temperature was reached. Electrolyte leakage measurement and total RNA isolation were performed with samples exposed to freezing conditions (0, −4, −8, −12, −16, and −20 °C), frozen in liquid nitrogen, and then stored at −80 °C, as illustrated in Figure 1.

### 4.3. Electrolyte Leakage Measurement

The electrolyte leakage percentage of *A. turgidum* (NA and CA) exposed to different freezing conditions (0, −4, −8, −12, −16, and −20 °C) was measured as previously described, with minor modification [78]. Samples were removed when they reached their designated temperatures and then immediately placed on ice to allow gradual thawing. After complete thawing, 2.4 mL DW was added to the tubes, followed by overnight shaking at room temperature. The conductivity of the solution in each tube was measured with a conductivity meter B-173 (Horiba, Japan). The tubes were then autoclaved. After shaking at room temperature, the conductivity of the solution in each tube was measured again. Finally, the percentage of electrolyte leakage was calculated as the percentage of the conductivity before autoclaving divided by that after autoclaving [79].

### 4.4. Chlorophyll Fluorescence Measurement

Immediately after freezing, *A. turgidum* plants were transferred to a BCDAT medium in a 25 °C culture room. Following 2 and 5 days of recovery, the maximum photochemical efficiency of photosystem II (calculated as F_V_/F_M_, where F_M_ is the maximum fluorescence of the dark-adapted plants under the light-saturating flash, and F_V_ is the maximum variable fluorescence, F_M_ − F_0_) was measured with samples dark-adapted for 30 min, using IMAGING-PAM (Walz, Effeltrich, Germany).

### 4.5. RNA Extraction and Library Preparation and Sequencing

To determine the transcriptome of *A. turgidum* protonema depending on whether or not cold acclimation treatment was undertaken, the total RNA of each sample (NA treated with −12 °C freezing stress; NA-12 vs. CA treated with −12 °C freezing stress; CA-12) was isolated using the RNeasy Plant Mini Kit (Qiagen, Hilden, Germany) according to the manufacturer’s instructions and then RNA was treated with RNase-Free DNase I (Takara, Japan) to remove any possible DNA. The integrity and concentration of RNA were determined using a Bioanalyzer (RIN > 6) (Agilent Technologies, Santa Clara, CA, USA) and a Qubit RNA Broad-Range Assay Kit (Life Technologies, Carlsbad, CA, USA), respectively. To construct the sequencing library, 1.5 μg of total RNA from each sample was used as the input for the TruSeq RNA sample prep kit v2 (Illumina, San Diego, CA, USA) following the manufacturer’s recommended method. In total, six libraries composed of three biological replicates of NA-12 and CA-12 samples were validated and then quantified using the Bioanalyzer and the library qPCR quantification method. These libraries were paired-end sequenced on a MiSeq using a MiSeq Reagent Kit v3 (2 × 300 bp) (Illumina). A total of 5 Gb of sequence data were generated (Q30 > 93%). The RNA-Seq data were deposited into the Sequence Read Archive under accession numbers SRS16292302 and SRS16292303.

### 4.6. De Novo Transcriptome Sequence Assembly and Functional Annotation

To improve the accuracy of the analysis, the quality check of the raw sequence data obtained after sequencing was performed using the fastQC (v0.11.1; http://www.bioinformatics.babraham.ac.uk/projects/fastqc/) and Trimmomatic (v0.33; http://www.usadellab.org/cms/?page=trimmomatic), with adaptor sequences and low-quality, reads removed. Filtered reads were merged to perform de novo assembly using Trinity (v2.1.1; http://trinityrnaseq.github.io/) with default parameters. Using TrinityStats, Transdecoder.LongOrfs (https://transdecoder.github.io/), and the CD-HIT-EST v4.6 (https://github.com/weizhongli/cdhit/blob/master/doc/cdhit-user-guide.wiki), assembled reads were made into high-quality, unique transcripts. The resulting unigene sequences were compared against the plant non-redundant (nr) protein database at the National Centre for Biotechnology Information (NCBI) using BLASTP with an E-value parameter not greater than 1 × 10^−4^ for identification of the best significant match. The BLASTP results were then imported into Blast2GO v3.1 [80] to retrieve the Gene Ontology (GO) terms of the assembled unigenes, and the annotation was further continued with unique enzyme codes (EC) and Kyoto Encyclopedia of Genes and Genomes (KEGG) maps. Moreover, the KEGG Automated Annotation Server (KAAS) was used for pathway mapping in addition to Blast2GO. GO terms are precisely defined as controlled vocabulary which can be used to describe the functions of genes or gene products. The assembled transcripts based on the retrieved GO terms were classified into three categories: biological processes, molecular functions, and cellular components. Pathway maps were determined from the KEGG database with an E-value of 1 × 10^−5^.

### 4.7. RNA-Seq Analysis

The high-quality trimmed raw reads were mapped to an assembled reference transcriptome by the HISAT v2.0.0 (Bowite2 v2.2.3; https://ccb.jhu.edu/software/hisat2/index.shtml) with default parameters, and the number of mapped reads were calculated by samtools v1.2 (http://www.htslib.org/). Differentially Expressed Genes (DEGs) were evaluated using Cuffdiff to compare treatment conditions. Unigenes with *p*-values < 0.05 and log_2_ fold-changes > 1 (i.e., fold-change > 2) were considered to be differentially expressed. A further screening of initial DEGs was based on fragments per kilobase per million fragments mapped (FPKM) values. The selected DEGs were used to create a heatmap using the Multi Experiment Viewer (MeV v.4.9.0) software tool [81].

### 4.8. Starch and Sugars Concentration Analysis

The starch extraction was performed as previously described [82]. Briefly, each sample was treated with 0.7 M perchloric acid. After centrifugation at 16,000× *g* for 5 min at room temperature, the supernatant was removed; the pellet was washed twice with 80% (*v*/*v*) ethanol and dried. DMSO was added, and the sample was incubated at 100 °C for 20 min to disperse the starch. The starch content was measured using Lugol’s solution at 620 nm. The absorbances of the sample were converted to concentration (ug/mL) using a standard curve of soluble starch (48425-1501, Junsei, Tokyo, Japan). To measure the soluble sugar content, the method described in previous research [83] was partially modified and used. One milliliter of 80% (*v*/*v*) methanol (MeOH) was added to 200 mg of the homogenized sample and mixed vigorously. Then, the mixed solution was incubated at 90 °C for 10 min and centrifuged at 16,000× *g* for 5 min. The supernatant was transferred to a new test tube, and the extraction process was repeated. The collected extract was lyophilized, dissolved in DW, and analyzed using HPLC with a Quaternay pump (flow rate: 0.5 mL/min), RI detector (Ultimate 3000; Dionex, Sunnyvale, CA, USA), and Aminex 87P ion exclusion column (10 µm, 7.8 × 300 mm). Distilled water was used as a mobile phase. The content of each detected sugar was calculated using the peak area of the sugar standards (maltose, sucrose, glucose, fructose) analyzed by HPLC (Figure S2).

### 4.9. Statistical Analysis

Data were presented as the mean ± standard deviation. Statistical analyses were performed using Student’s t-test in Microsoft excel (* *p* < 0.05; ** *p* < 0.01; and *** *p* < 0.001).

## 5. Conclusions

Svalbard, a Norwegian archipelago, has a typical Arctic environment. Summer in the archipelago lasts 3.5 months, from mid-May to September, with an average temperature of 5.8 °C. Winter lasts 8.5 months, from September to mid-May of the following year, and has an average temperature of −12 °C. The overall Arctic atmosphere is dry [84], though atmospheric humidity is relatively higher in summer than in winter. Here, we used *Aulacomnium turgidum* (Wahlenb.) Schwaegr, a moss found in this region, to investigate cold acclimation and freezing tolerance through de novo transcriptome assembly and RNA-seq analysis. AP2/ERF family transcription factors and PPR protein genes involved in carbohydrate metabolism were identified as DEGs induced by cold acclimation under freezing stress. In addition, it was found that the concentration of starch and maltose in *A. turgidum* increased with cold acclimation. The unigenes and DEGs associated with cold acclimation identified in this study provide a foundation for further studies on the mechanisms behind freezing stress regulation in *A. turgidum* and other non-seed plant species. Furthermore, lineage-specific stress-regulated genes not found in flowering plants could be important clues for protecting crops in a climate change caused by global warming.

## Figures and Tables

**Table 1:** Sequencing output statistics of *A. turgidum* from this study.

Accession	No. Raw Reads	No. Trimmed Reads	% of Reads after Trimming	Size of Trimmed Reads (bp)	GC (%)	Q30 (%)
NA-12_1	3,529,180	3,305,320	93.66	814,851,894	49.13	93.77
NA-12_2	3,759,002	3,536,726	94.09	879,302,307	49.63	93.84
NA-12_3	3,446,114	3,202,404	92.93	789,298,951	49.5	93.61
CA-12_1	3,636,552	3,421,010	94.07	848,381,538	49.24	93.8
CA-12_2	3,241,544	3,006,906	92.76	732,304,413	49.22	93.52
CA-12_3	3,727,552	3,518,610	94.39	874,412,690	49.66	93.86

**Table 2:** Summary of contigs and unigenes.

	Contig	Unigene
Total Length (bp)	157,581,303	24,753,852
No. Sequence	116,979	45,796
Max. Length (bp)	19,624	17,139
Mean Length (bp)	1347	541
N50 (bp)	2481	1017
N90 (bp)	507	207
N90 Sequence No.	73,457	31,863
GC%	46.5	49.2

**Table 3:** KEGG analysis of DEGs upregulated in CA-12.

Class	Pathway	ID	DEG	All Genes	*p*-Value	Corrected *p*-Value
**Category/Metabolism**						
Carbohydrate metabolism	Starch and sucrose metabolism	ppp00500	32	198	1.46 × 10^−11^	4.57 × 10^−10^
	Glycolysis/gluconeogenesis	ppp00010	26	172	4.06 × 10^−9^	7.63 × 10^−8^
	Amino sugar and nucleotide sugar metabolism	ppp00520	23	137	5.49 × 10^−9^	8.61 × 10^−8^
	Galactose metabolism	ppp00052	13	52	2.32 × 10^−7^	3.12 × 10^−6^
	Pentose phosphate pathway	ppp00030	16	86	3.36 × 10^−7^	3.91 × 10^−6^
	Butanoate metabolism	ppp00650	9	24	1.06 × 10^−6^	9.93 × 10^−6^
	Fructose and mannose metabolism	ppp00051	12	88	1.46 × 10^−4^	7.64 × 10^−4^
	Ascorbate and aldarate metabolism	ppp00053	8	52	9.09 × 10^−4^	3.88 × 10^−3^
	Pyruvate metabolism	ppp00620	12	111	9.86 × 10^−4^	4.03 × 10^−3^
	Glyoxylate and dicarboxylate metabolism	ppp00630	11	107	2.27 × 10^−3^	8.54 × 10^−3^
Amino acid metabolism	Phenylalanine metabolism	ppp00360	12	45	3.74 × 10^−7^	3.91 × 10^−6^
	Alanine, aspartate, and glutamate metabolism	ppp00250	12	59	4.38 × 10^−6^	3.74 × 10^−5^
	Tyrosine metabolism	ppp00350	8	43	2.96 × 10^−4^	1.39 × 10^−3^
Lipid metabolism	Fatty acid biosynthesis	ppp00061	11	60	2.56 × 10^−5^	2.01 × 10^−4^
	Glycerophospholipid metabolism	ppp00564	13	87	3.37 × 10^−5^	2.43 × 10^−4^
	Cutin, suberine, and wax biosynthesis	ppp00073	5	11	1.41 × 10^−4^	7.64 × 10^−4^
Biosynthesis of other secondary metabolites	Phenylpropanoid biosynthesis	ppp00940	15	117	4.34 × 10^−5^	2.84 × 10^−4^
Metabolism of other amino acids	Taurine and hypotaurine metabolism	ppp00430	5	8	4.54 × 10^−5^	2.84 × 10^−4^
	beta-Alanine metabolism	ppp00410	6	36	2.76 × 10^−3^	9.97 × 10^−3^
Energy metabolism	Carbon fixation in photosynthetic organisms	ppp00710	15	125	8.56 × 10^−5^	5.03 × 10^−4^
Metabolism of cofactors and vitamins	Ubiquinone and other terpenoid-quinone biosynthesis	ppp00130	8	40	1.92 × 10^−4^	9.48 × 10^−4^
**Category/Cellular Processes**						
Transport and catabolism	Peroxisome	ppp04146	11	96	1.03 × 10^−3^	4.02 × 10^−3^

## Data Availability

The data that support the findings of this study are openly available in GenBank of NCBI at https://www.ncbi.nlm.nih.gov. The BioProject, SRA, and Bio-Sample numbers are PRJNA916379, SRS16292302, SRS16292303, SAMN32423464, and SAMN32423465, respectively.

## Supplementary Materials

The following supporting information can be downloaded at: https://www.mdpi.com/article/10.3390/plants12061250/s1. Table S1. Detailed information on non-redundant database blast results. Table S2. Gene ontology (GO) assignments in the categories of biological processes, molecular functions, and cellular components for whole unigenes. Table S3. KEGG pathway analysis in the cellular processes, environmental information processing, genetic information processing, metabolism, and organismal systems for whole unigenes. Table S4. List of differentially expressed genes between NA-12 and CA-12. Table S5. Primer sequences used for real-time RT-PCR analyses. Table S6. Detailed information for Gene Ontology (GO) assigned to unigenes upregulated in CA-12. Table S7. Detailed information of KEGG pathway analysis assigned to unigenes upregulated in CA-12. Figure S1. Lists of unigenes related to the synthesis, decomposition, conversion, and transport of starch, maltose, glucose, fructose, and sucrose. Figure S2. Chromatograms of the sugar standards, NA-12 and CA-12 using HPLC-RI.
