# Supplementary material for: De Novo Transcriptome Assembly and Comparative Analysis of Differentially Expressed Genes Involved in Cold Acclimation and Freezing Tolerance of the Arctic Moss Aulacomnium turgidum (Wahlenb.) Schwaegr
Source: Plants (Basel). 2023 Mar 9;12(6):1250. doi: 10.3390/plants12061250 (PMC10054522; doi:10.3390/plants12061250)
Supplement: Supplementary file 1 [file plants-12-01250-s001.zip › Plants-2162636.Supplementary.Figure.v6.2.pdf]

(A) Starch and maltose

Function	Enzyme name	Unigene No.	Up-regulated unigene from CA		
			Average of expression value		log2 Fold Change
			NA-12	CA-12	
Synthesis	SS	unigene_02205	0.18	5.31	5.13
		unigene_14682	5.13	32.66	2.88
	SBE	unigene_11066	30.71	130.18	2.29
Decomposition	GWD	unigene_13228	4.59	19.56	2.32
		unigene_34485	58.58	750.75	3.89
	αAM	unigene_13443	6.90	1462.61	7.94
		unigene_24164	7.09	149.12	4.61
	βAM	unigene_15306	160.24	1274.82	3.20
		unigene_41589	39.69	160.58	2.22

(B) Glucose

Function	Enzyme name	Unigene No.	Up-regulated unigene from CA		
			Average of expression value		log2 Fold Change
			NA-12	CA-12	
Decomposition	NIN	unigene_39873	42.62	234.22	2.67
	DPE1	unigene_05326	28.95	100.43	2.01
		unigene_23568	1.21	38.22	5.19
Transporter	GLUT	unigene_32381	39.64	160.31	2.22
		unigene_32594	6.30	75.68	3.81
		unigene_32989	1.17	42.28	5.37

(C) Sucrose

Function	Enzyme name	Unigene No.	Up-regulated unigene from CA		
			Average of expression value		log2 Fold Change
			NA-12	CA-12	
Synthesis	SPS	unigene_05471	16.40	134.84	3.25
ΨSyn. or Deco.	SuSy	unigene_34305	1.67	168.73	6.87
		unigene_39191	15.77	117.66	3.11
Conversion	SPP	unigene_22087	16.76	140.41	3.28
		unigene_23848	7.00	75.94	3.66
Transporter	SUT	unigene_05657	24.55	162.52	2.94
		unigene_07358	15.60	105.57	2.98
		unigene_19674	28.49	212.78	3.11
		unigene_22087	16.76	140.41	3.28
		unigene_23848	7.00	75.94	3.66
		unigene_37984	15.08	66.21	2.35
Decomposition	NIN	unigene_39873	42.62	234.22	2.67

(D) Fructose

Function	Enzyme name	Unigene No.	Up-regulated unigene from CA		
			Average of expression value		log2 Fold Change
			NA-12	CA-12	
Decomposition	NIN	unigene_39873	42.62	234.22	2.67
Transporter	SDH	unigene_16245	0.41	9.24	4.69
		unigene_28068	9.24	34.89	2.12
Conversion	FK	unigene_01128	28.92	104.95	2.07
		unigene_25770	16.63	47.92	1.75
		unigene_34353	11.90	54.30	2.40
		unigene_43437	2.54	27.03	3.61
ΨSyn. or Deco.	SuSy	unigene_34305	1.67	168.73	6.87
		unigene_39191	15.77	117.66	3.11

Figure S1. Lists of unigenes related to the synthesis, decomposition, conversion, and transport of starch, maltose, glucose, fructose, and sucrose. In the $\log_2$ fold change column, red indicates the highest value and white indicates the lowest value. CA-12, *A. turgidum* protonema were cold-acclimated at 4 °C for 2 days and exposed to freezing until -12 °C; NA-12, *A. turgidum* protonema were non-acclimated at 25 °C for 2 days and exposed to freezing until -12 °C.

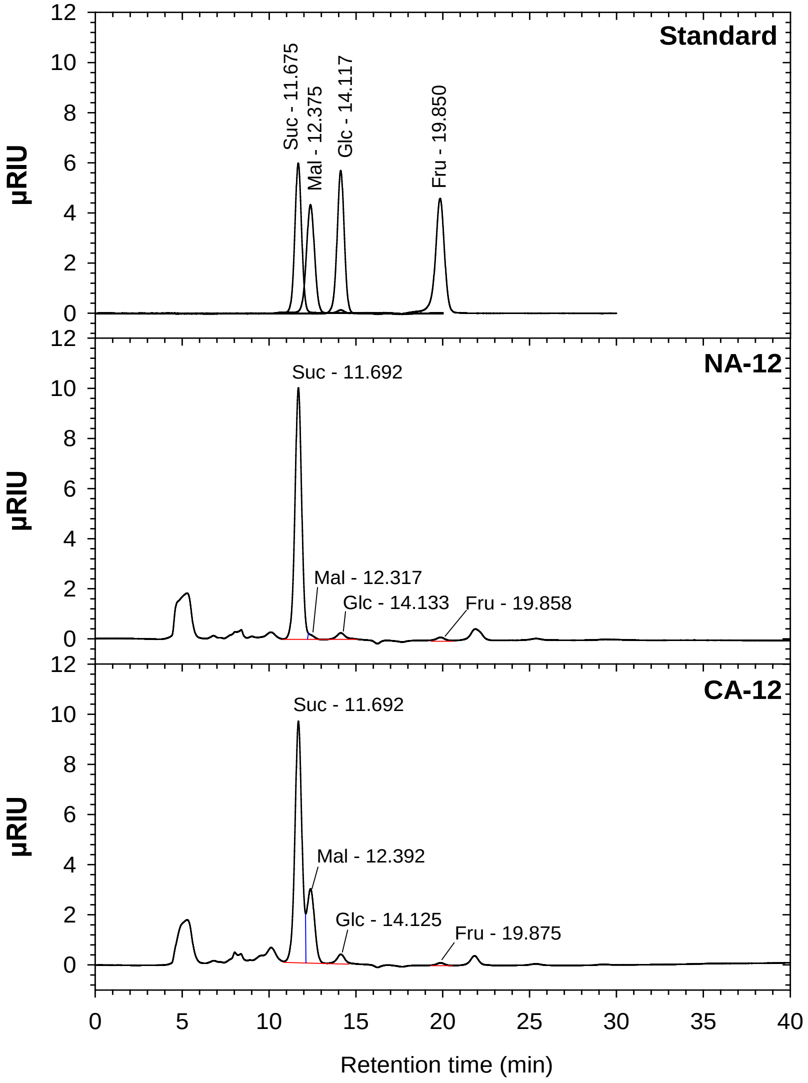

Figure S2. Chromatograms of sugars for standards, NA-12 and CA-12 using HPLC-RI. Soluble sugars were separated using a Aminex 87P ion exclusion column at a flow rate of 0.5 mL/min. Distilled water was used as a mobile phase. NA-12, *A. turgidum* protonema were non-acclimated at 25 °C for 2 days and exposed to freezing until -12 °C; CA-12, *A. turgidum* protonema were cold-acclimated at 4 °C for 2 days and exposed to freezing until -12 °C; Suc; sucrose, Mal; maltose, Glc; glucose, Fru; fructose.
